# *ESR1* fusion proteins in breast cancer: distinguishing oncogenic drivers from passenger events

**DOI:** 10.1038/s41388-026-03813-w

**Published:** 2026-05-14

**Authors:** Ana-Maria Gherghelas, Christopher P. Toseland

**Affiliations:** Divisional of Clinical Medicine, School of Medicine and Population Health, University of Sheffield, Sheffield, United Kingdom

**Keywords:** Nuclear organization, Breast cancer, Transcription

## Abstract

Breast cancer is characterised by profound genetic heterogeneity, with oestrogen receptor alpha (ERα, encoded by *ESR1*) being a central driver in ~70% of cases. While point mutations in the ligand-binding domain of *ESR1* are well recognised mediators of endocrine resistance, a growing body of evidence highlights *ESR1* gene fusions as an emerging class of genomic alterations with important clinical implications. These rearrangements, predominantly arising from intrachromosomal events on chromosome 6, truncate the hormone-binding domain and fuse *ESR1* with diverse partners, producing constitutively active chimeric proteins. Recurrent fusions such as *ESR1–CCDC170*, *ESR1–YAP1*, and *ESR1–AKAP12* have been consistently associated with therapy resistance, metastatic progression, and poor clinical outcome, suggesting that they function as oncogenic drivers rather than incidental by-products of genomic instability. However, variability in oncogenic potential indicates that only a subset of fusions are biologically functional. Key determinants of functionality include reading-frame preservation, domain architecture, intrinsic disorder content, and the molecular features contributed by the partner gene, many of which encode transcription factors, signalling adaptors, or cytoskeletal regulators. Structural enrichment in intrinsically disordered regions and the potential for phase separation further implicate *ESR1* fusions in the formation of aberrant transcriptional condensates that amplify ER signalling. Importantly, while many fusions are enriched in metastatic, treatment-resistant disease, a small number are present in treatment-naïve tumours, raising the possibility that some act as early drivers of oncogenesis. This review outlines the current knowledge on *ESR1* fusions, evaluating their mechanisms and clinical relevance. Clarifying which *ESR1* fusions act as true oncogenic drivers versus incidental events will be critical for refining diagnostics and informing future therapeutic development for oncofusion-driven cancers.

## Introduction

Breast cancer is a highly heterogeneous disease where point mutations and gene amplifications, are well-characterised drivers of tumour development. Advances in deep sequencing have uncovered novel genomic alterations, including gene fusions that generate hybrid proteins combining domains from distinct partners [1, 2]. The proteins produced by these chimeric transcripts often display altered expression, regulation and/or structure [3, 4], which can affect their original functionality and even introduce novel properties [5].

The prevalence of gene fusions varies across cancer types, with currently known translocations accounting for ~20% of cases [6]. Whilst extensively studied in haematological malignancies, their presence and pathological implication in solid tumours is poorly understood [6, 7]. However, the number of gene fusions detected in solid tumours has increased in recent years, suggesting that chromosomal events are more prevalent than previously recognised [1, 8].

In breast cancer, several recurrent gene fusions have been identified, including *ETV6–NTRK3*, *ERBB2*, *RET*, *FGFR*, *BRAF* and *MAST* rearrangements [9–12]. Among these, ESR1 fusions represent an emerging class of genomic alterations. ESR1 encodes oestrogen receptor alpha (ERα), which is expressed in ~70% of breast cancers and drives transcriptional programmes governing proliferation and survival. Aberrant ER signalling most commonly arises from ligand-binding domain (LBD) mutations that confer ligand-independent activity and endocrine resistance [12–14] A detailed overview of ER-activating mutations and their clinical implications is provided here [15, 16].

**Fig. 1:**
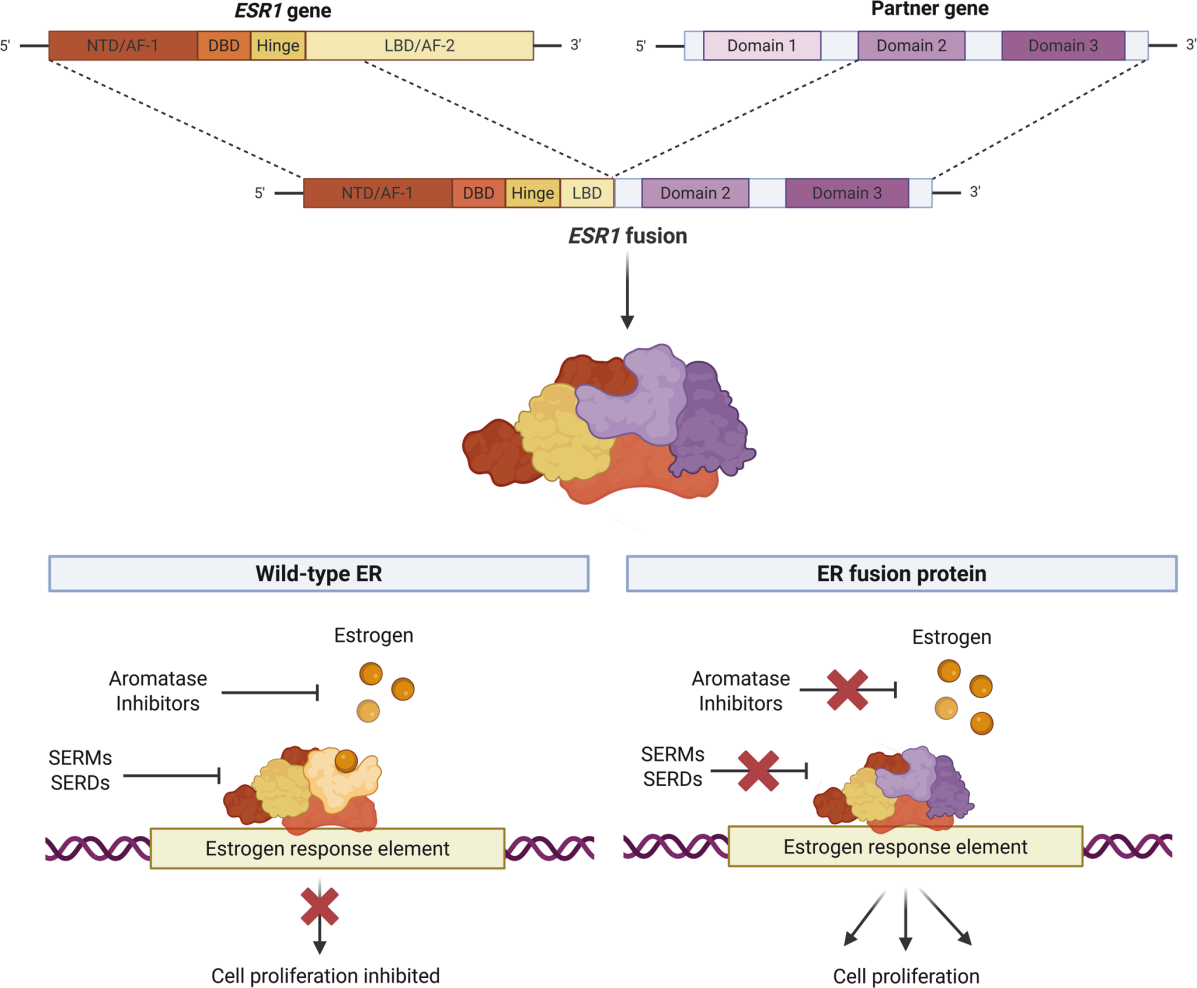
Domain architecture of *ESR1* and its alteration through gene fusion events. The wild-type *ESR1* gene encodes the oestrogen receptor α (ERα), which comprises four major functional domains. The N-terminal domain (NTD) regulates transcription in a ligand-independent manner via the Activation Function-1 (AF-1) domain. The central DNA-binding domain (DBD) binds to oestrogen response elements (EREs) to control target gene expression. This is followed by a flexible hinge region, containing the nuclear localization signal. The C-terminal ligand-binding domain (LBD) includes the hormone-binding site for ligands such as oestrogen and overlaps with the Activation Function-2 (AF-2) domain, which mediates ligand-dependent transcriptional activity. The LBD is the primary target of selective ER modulators (SERMs) and degraders (SERDs). During a fusion event, *ESR1* joins with another gene, producing a chimeric protein that retains functional domains from both gene partners. In most ER fusion proteins, the LBD is truncated, preventing oestrogen, SERMs, or SERDs from binding. Consequently, the fusion protein is constitutively active and drives transcription in a hormone-independent manner. Illustration created on BioRender.com [14, 19, 28, 29, 31].

ESR1 fusions are predominantly identified in endocrine-resistant disease. These rearrangements typically truncate the LBD and fuse the N-terminal portion of ESR1 to diverse partners, generating chimeric proteins with ligand-independent transcriptional activity (Fig. 1). Consequently, they are associated with multiple pro-tumourigenic processes. [14, 17–22] See Table 1.

**Table 1:** Functional classification of ESR1 fusion events in breast cancer.

Strong evidence of oncogenic potential					
*ESR1* fusion partner	Structural characteristics (*ESR1* exon, frame)	Chromosome of partner gene	Clinical characteristics/ therapy exposure	Oncogenic function	References
*ARNT2*	Exon 6 (in-frame)	15 (inter)	Metastatic ER+	Drives oestrogen-independent, fulvestrant-resistant growth Increases motility, migration and invasion in T47D cells Induces ER target genes (*GREB1, TFF1, PGR*) and EMT-related genes (SNAI1)	[20]
*CCDC170*	Exon 2 (5’UTR-CDS) Exon 4 (out-of-frame) Exon 5 (out-of-frame)	6 (intra)	Primary, treatment-naïve Luminal B	Isoform-dependent reduced endocrine sensitivity in vivo Enhances cell migration, anchorage-independent growth and colony formation Activates GAB1-AKT/ERK signalling	[18, 32]
*PCDH11X*	Exon 6 (in-frame)	X (inter)	Advanced, endocrine-resistant ER+	Fulvestrant-resistant growth, higher cell migration and invasion in a hormone-independent manner Induces ER target genes (*GREB1*, *TFF1*, *PGR*) and EMT-related genes (SNAI1), leading to higher cellular motility and metastatic capacity to the lung in xenograft mouse models	[19, 20]
*SOX9*	Exon 6 (in-frame)	17 (inter)	Metastatic ER+ (liver metastasis)	Stable ESR1 fusion proteins that confer ligand-independent activity Considered hyperactive, resulting in ER activity 40 higher than basal wild-type ER activity Drove significantly higher growth than YFP control cells in presence of fulvestrant, and induced higher cell migration and invasion than controls in a hormone-independent manner Induces ER target genes (*GREB1, TFF1, PGR*) and EMT-related genes (SNAI1)	[20, 28]
*YAP1*	Exon 6 (in-frame)	11 (inter)	Pan-endocrine therapy resistant, metastatic ER+	Hyperactive ER fusion (40× basal ER activity) Oestrogen-independent cell growth, migration and invasion Fulvestrant-resistant growth, and higher proliferation in low-oestrogen conditions Induces ER target genes (*GREB1, TFF1, PGR*) and EMT-related genes (*SNAI1*), leading to higher cellular motility and metastatic capacity to the lung in xenograft mouse models	[20, 28, 29, 34]
Partial/context-dependent evidence of oncogenic potential					
*CLINT1*	Exon 6 (in-frame)	5 (inter)	Metastatic ER+	Enhances endocrine-resistant growth and oestrogen-independent motility	[20]
*DAB2*	Exon 6 (in-frame)	5 (inter)	Endocrine-resistant, metastatic ER+ (supraclavicular lymph node metastasis)	Hyperactive ER fusion (40x basal ER activity) Oestrogen-independent, fulvestrant-resistant growth in MCF7 cells, but not in T47D cells (cell line-dependent) Lack of oestrogen-independent activation of ERα target genes and EMT-related genes in T47D cells	[28, 29]
*GRIP1*	Exon 6 (in-frame)	12 (inter)	Metastatic ER+	Enhances endocrine-resistant growth and oestrogen-independent motility	[20]
*GYG1*	Exon 6 (in-frame)	3 (inter)	Endocrine-resistant, metastatic ER+ (bone metastasis)	Ligand-independent activity without activation of ERα target genes and EMT-related genes in T47D cells	[20, 28]
*LPP*	Exon 6 (in-frame)	3 (inter)	Metastatic ER+	Promotes oestrogen-independent, fulvestrant-resistant growth and motility in T47D cells	[20]
*NCOA1*	Exon 6 (in-frame)	2 (inter)	Metastatic ER+	Promotes oestrogen-independent, fulvestrant-resistant growth and motility in T47D cells	[20]
*TNRC6B*	Exon 6 (in-frame)	22 (inter)	Metastatic ER+	Enhances oestrogen-independent growth and motility	[20]
Inactive/unknown functions					
*ARID1B*	Exon 6 (in-frame)	6 (intra)	Metastatic ER+	Stable chimeric proteins but transcriptionally inactive with no growth or metastatic phenotypes in T47D cells	[20]
*NOP2*	Exon 6 (in-frame)	12 (inter)	Primary, treatment-naïve	Stable chimeric proteins but transcriptionally inactive with no growth or metastatic phenotypes in T47D cells	[19, 29]
*PCMT1*	Exon 6 (in-frame)	6 (intra)	Metastatic ER+	Stable chimeric proteins but transcriptionally inactive with no growth or metastatic phenotypes in T47D cells	[20]
*TCF12*	Exon 6 (in-frame)	15 (inter)	Metastatic ER+	Stable chimeric proteins but functionally inactive in both T47D and MCF7 cells	[20]
*CDK13*	Exon 7 (in-frame)	7 (inter)	Stage IV, endocrine-resistant (ctDNA)	Unknown	[28]
*MTHFD1L*	Exon 7 (in-frame)	6 (intra)	Late-stage, endocrine-resistant	Unknown	[28]
*ARMT1*	Uknown	6 (intra)	Treatment-naïve ER+, Luminal A-like subtype	Unknown	[30]
*POLH*	Exon 7 (in-frame)	6 (intra)	Primary ER+	Unknown	[19]
*AKAP12*	Exon 6 (out-of-frame)	6 (intra)	Endocrine-resistant, metastatic ER⁺	Unknown	[28, 37]
*AKR1D1*	Exon 6 (out-of-frame)	7 (inter)	Primary, treatment-naïve, ER+	Unknown	[19]
*COA5*	Exon 4 (in-frame)	2 (inter)	Metastatic ER+	Unknown	[22]
*NKAIN2*	Exon 6 (in-frame)	6 (intra)	Stage IV, endocrine-resistant (ctDNA)	Unknown	[28]
*PLEKHG1*	Exon 6 (in-frame)	6 (intra)	Metastatic ER+	Unknown	[21]
*TFG*	Exon 6 (in-frame)	3 (inter)	Metastatic ER+	Unknown	[28]

*ESR1* fusions categorised into functionally distinct groups ranging from potent oncogenic drivers to transcriptionally inactive or uncharacterised events. Strong oncogenic fusions consistently drive ligand-independent ER signalling, endocrine therapy resistance, and metastatic phenotypes, whereas other fusions exhibit context-dependent or no detectable functional activity despite stable expression.

Despite their emerging clinical relevance, the molecular mechanisms of ESR1 fusions remain poorly defined, reflecting the broader challenge of distinguishing oncogenic drivers from by-products of genomic instability [23]. Most gene fusions are rare and structurally diverse, arising from stochastic rearrangements that often generate non-functional products [23]. Indeed, over 80% of the nearly 10,000 fusion transcripts identified to date are associated with genomic instability [2, 22] and are considered passenger events with little impact on tumourigenesis [22, 23].

Nevertheless, recurrent fusions are often enriched in specific cancer subtypes, disrupt key regulatory pathways, or encode gain-of-function proteins, suggesting strong selective pressure [2, 5, 22–24]. Fusion events can occur with few additional mutations, suggesting that a single rearrangement may be sufficient to initiate malignant transformation [23]. However, the detection of fusion transcripts in non-malignant tissues further challenges their implication in neoplastic transformation, highlighting the need to distinguish true oncogenic drivers from incidental passenger events [6].

In this review, we use ESR1 fusions as a model system to examine how structural features, recurrence patterns, and functional consequences inform oncogenic potential, with the aim of clarifying general principles that distinguish driver fusions from passengers and identifying key unresolved questions in the field.

## Recurrent *ESR1* fusions and their role in therapy resistance

To date, ~30 distinct *ESR1* fusion variants have been reported, with *ESR1-CCDC170*, *ESR1-YAP1* and *ESR1-AKAP12* being among the most frequently observed. Although some have only been identified in individual samples, the majority of *ESR1* fusions have been detected in multiple patients, highlighting their recurrent nature. Their prevalence is currently estimated at 1–10% of breast cancer cases [18, 20, 25]. As genomic profiling improves, detection rates are likely to increase, clarifying their prevalence and significance in disease.

*ESR1* fusions have been predominantly identified in ER-positive tumours (ER+), which highly express oestrogen receptors and rely on oestrogen signalling for growth [17, 26, 27]. They have been detected across both luminal A and luminal B subtypes but are rare in ER negative (ER-) and triple-negative breast cancers (TNBC), which generally lack ER expression [17, 20]. This pattern implies that *ESR1* overexpression may be critical for fusion formation, potentially by increasing the likelihood of transcription–replication conflicts, which can lead to DSBs. Notably, *ESR1* fusions have not been reported in healthy individuals, although it remains unclear whether this reflects true absence or limited screening. This also questions the suitability of standard detection techniques like immunohistochemistry for detecting *ESR1* fusions in ER+ tumours. These methods typically assess overall ER expression but are not designed to detect *ESR1* rearrangements, highlighting the need for more targeted sequencing approaches.

Currently identified *ESR1* fusions are markedly enriched in metastatic, treatment-resistant cases [17, 20, 26, 28]. This resistance is largely driven by the truncation of the *ESR1* LBD – a critical region targeted by most endocrine-based therapies (Fig. 1) [18, 26, 29]. Multiple fusions, including *ESR1–YAP1*, *ESR1–PCDH11X*, *ESR1–AKAP12*, *ESR1–CCDC170*, and *ESR1–SOX9*, confer resistance to oestrogen deprivation and selective ER modulators or degraders, such as tamoxifen and fulvestrant [29, 30]. Several other fusions such as *ESR1-ARNT2*, *ESR1-LPP, ESR1-NCOA1*, *ESR1-CLINT1*, *ESR1-GRIP1*, and *ESR1-TNRC6B* also promote robust cell proliferation and motility in vitro despite endocrine treatment, highlighting their capacity to sustain tumour growth in a hormone-independent and treatment-resistant manner [19].

Beyond therapeutic resistance, loss of the LBD is associated with constitutive, ligand-independent ER signalling resulting in the hyperactivation of downstream pathways [29]. For example, *ESR1-YAP1*, *ESR1-PCDH11X*, *ESR1-SOX9*, *ESR1-ARNT2*, *ESR1-LPP*, *ESR1-NCOA1* and *ESR1-DAB2* promote oncogenic traits such as increased cell proliferation, motility, and invasion in vitro [15–19, 31]. This may be attributed to the oestrogen-independent expression of canonical ER target genes, including *GREB1*, *TFF1*, and *PGR* [18, 19]. In vivo and xenograft models further demonstrate that fusions such as *ESR1–YAP1* and *ESR1–PCDH11X* enhance expression of epithelial-to-mesenchymal transition (EMT)–associated genes, including *SNAI1*, thereby promoting invasive and metastatic phenotypes [18, 19]. Notably, *ESR1-DAB2* and *ESR1-SOX9* have been reported to increase ER activity by up to 40-fold relative to wild-type ER [26]. This sustained signalling is likely facilitated by the activity of overexpressed *ESR1* fusion proteins that ultimately make tumours less responsive to treatment [18–20]. However, it remains unclear whether these effects are mediated exclusively through aberrant ER signalling. Additional mechanisms including altered protein–protein interactions, engagement of pathways associated with the fusion partner, or novel biophysical properties such as phase separation may be contributing to the oncogenic potential of these fusion proteins, although direct evidence for these is currently limited and requires further investigation. Interestingly, a small number of *ESR1* fusions have been detected in primary and/or treatment-naive breast tumours, such as *ESR1-e2* > *CCDC170*, *ESR1-e4* > *CCDC170*, *ESR1-e5* > *CCDC170*, *ESR1-e6* > *NOP2*, *ESR1-e6* > *AKR1D1* and *ESR1-e6* > *POLH* [17, 18, 32]. Determining the timing of their emergence during tumour evolution remains a critical question, as some of these fusions might arise early in tumour development and act as initial drivers of cancer formation. This emphasises the need for sensitive diagnostic tools and routine screening to enable earlier detection and more effective disease management.

## The role of spatial proximity in *ESR1* fusions

In breast cancer, gene fusions are predominantly intrachromosomal, involving rearrangements between genes located on the same chromosome [33, 34]. *ESR1* fusions follow the same pattern, with many arising from duplications and translocations within the *ESR1* locus on chromosome 6. The most well-characterised example is the tandem duplication between *ESR1* and the adjacent gene *CCDC170*, which accounts for a large proportion of *ESR1* fusion cases [17, 20, 28, 31, 35]. Interchromosomal rearrangements of *ESR1* have also been detected, involving genes located on entirely different chromosomes, such as *ESR1-PCDH11X*, *ESR1-SOX9* and *ESR1-ARNT2*. Although gene proximity appears to be favoured in fusion formation [2, 6], the occurrence of interchromosomal rearrangements suggests that additional factors contribute to *ESR1* fusion events. This raises key questions: what drives the selective fusion of *ESR1* with specific gene partners, and what enables rearrangements to occur across chromosomes despite the genomic distance?

One possible explanation lies in the three-dimensional architecture of chromosomes within the nucleus. The non-random distribution of chromosomes is primarily influenced by their size and gene density [36]. Smaller, gene-rich chromosomes are typically located closer to the centre of the nucleus, while larger, gene-poor chromosomes tend to be found in the nuclear periphery [36, 37]. This nuclear organisation brings certain chromosomes and genes into close spatial proximity, predisposing them to erroneous end-joining when DSBs occur [36, 38–40]. This principle underlies the contact-first model, which proposes that loci already positioned near one another are more likely to undergo translocations, supporting the role of nuclear architecture in the frequency and fusion partner selection [41, 38–40,42, 43].

However, spatial proximity alone cannot explain the full diversity of fusion patterns. The breakage-first model provides an alternative, suggesting that chromosome ends can move within the nucleus to locate compatible partners, thereby enabling translocations even between distant loci [36, 39, 43]. Although less efficient, such long-range movements plausibly account for rare but functionally important interchromosomal *ESR1* fusions. These may be facilitated by chromatin looping or cancer-associated changes in nuclear architecture that juxtapose genomic regions normally kept apart [36].

Beyond spatial positioning, chromatin accessibility and transcriptional activity may also influence the occurrence of fusion events. Regions of open chromatin are more susceptible to DSBs, increasing the risk of translocation events [44]. Nuclear architecture may be re-shaped by ligand-activated nuclear receptors, which can bring together transcriptionally active regions and promote site-specific DNA breaks that facilitate recurrent oncogenic translocations. Therefore, it is plausible that sustained ER signalling increases chromatin accessibility and DNA break susceptibility at the *ESR1* locus, predisposing it to non-random rearrangements with other actively transcribed fusion partners.

In parallel, higher rates of transcription are associated with increased replication stress, thus predisposing genomic fragile sites to DNA breaks [45]. Actively transcribed genes often co-localise in regions known as transcription factories, where multiple genes are clustered to enhance transcription efficiency [41, 46, 47]. This increases the chances of coincidental fusion during repair of transcription-associated DSBs. As a result, highly transcribed genes like *ESR1* may be more prone to transcription–replication conflicts, further increasing their susceptibility to forming fusions with other actively transcribed genes found within the same nuclear regions. Collectively, *ESR1* fusions are likely driven by a combination of spatial, structural, and functional factors. Intrachromosomal rearrangements are often facilitated by local proximity within chromosome 6, whereas fusions between distant chromosomes may arise from their nuclear arrangement or large-scale chromatin movements following DNA damage. Although interchromosomal translocations are structurally complex rearrangements, the recurrence of certain *ESR1* interchromosomal fusions may reflect evolutionary selection rather than random chance, highlighting their biological significance. Therefore, understanding the interplay between genome organisation, chromatin state, and transcriptional activity will be essential for revealing patterns of recombination that may help distinguish driver from passenger fusions.

## Reading frame compatibility is a crucial determinant of *ESR1* fusion functionality

Despite the functional consequence of many *ESR1* fusions, a subset appears to lack oncogenic activity and may instead represent passenger events. For instance, *ESR1-NOP2*, *ESR1-TCF12*, and *ESR1-GYG1* do not induce endocrine resistance, growth or metastatic traits in vitro [18, 19]. Similarly, certain *ESR1-CCDC170* fusion variants are functionally inactive or display variable sensitivity to endocrine treatments, while others exhibit clear oncogenic activity that contributes to increased proliferation, migration and invasion in vitro [17, 18, 30]. This variability prompts the question: why do some *ESR1* fusions produce functional, oncogenic proteins while others remain inactive or biologically neutral?

A major determinant of fusion functionality is whether the rearrangement results in a stable chimeric product. Reading frame compatibility heavily influences the ability to translate an amino acid sequence into functional proteins. Fused genes that maintain an open reading frame are classified as in-frame fusions, retaining the ability to produce stable, chimeric products [48]. These are significantly more prevalent and often result in proteins with novel or gain-of-function activity [48]. Contrastingly, out-of-frame fusions are typically not translated into functional proteins due to the presence of a premature stop codon or frameshift [48, 49]. Although not detrimental, in rare cases, they may still cause harmful effects if the affected gene is a tumour suppressor.

**Fig. 2:**
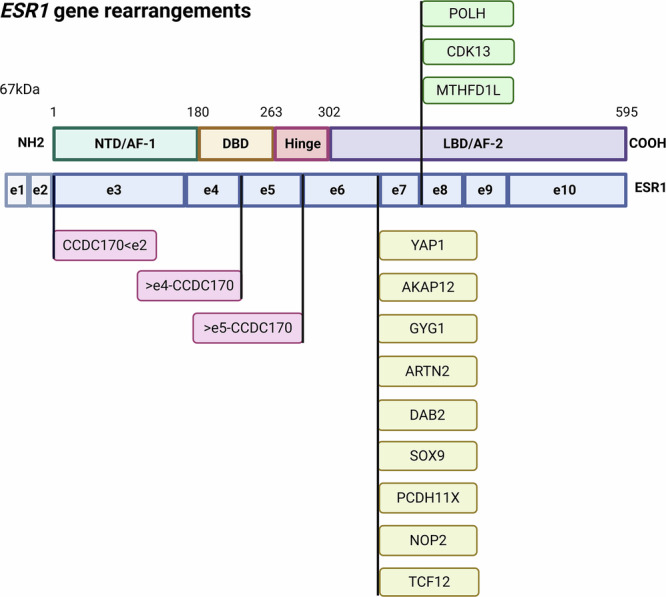
Examples of genomic rearrangements involving the *ESR1* gene. The *ESR1* gene consists of ten exons, of which exons 3–10 encode the major functional domains of the oestrogen receptor α (ERα). Most gene partners fuse with exon 6 or exon 7 of the *ESR1*, resulting in the loss of a large portion of the ligand-binding domain (LBD) and activation function-2 (AF-2) domain, while preserving all other upstream domains. The *CCDC170* gene has been shown to fuse with the *ESR1* gene at multiple sites, including exon 2, 4 and 5, indicating heterogeneity in fusion breakpoints and generating chimeric proteins with distinct structural and functional properties. Illustration created on BioRender.com [18, 19, 29, 31, 33].

Genomic rearrangements can be further classified as balanced, involving no net gain or loss of genetic material, or unbalanced, which are associated with copy-number alterations such as deletions or duplications [42]. Notably, despite their distinct origin, both balanced and unbalanced events can generate functional *ESR1* fusions proteins when breakpoints preserve the DBD while truncating the LBD, indicating that domain retention rather than balance status is the dominant determinant of oncogenic potential. Consistent with this, we calculate that nearly 80% of *ESR1* fusions are in-frame, with the fusion breakpoint frequently occurring after exon 6 (Fig. 2) [27, 29], preserving the DBD while truncating most, if not all, of the LBD. These chimeric proteins often exhibit constitutive, ligand-independent ER signalling [15, 27, 29], which directly contributes pro-tumourigenic transcriptional programmes. Our calculations further show that nearly 70% of *ESR1* fusions follow this breakpoint pattern, indicating a recurrent site of genomic rearrangement and reinforcing the importance of reading-frame preservation for fusion functionality. Most ESR1 fusions arise from unbalanced intrachromosomal rearrangements, such as deletions or tandem duplications associated with local copy-number changes, exemplified by ESR1–CCDC170 at the 6q25 locus [27, 29]. In contrast, fusions such as ESR1–YAP1 and ESR1–PCDH11X result from interchromosomal balanced rearrangements [18] but similarly generate oncogenic, in-frame chimeras. Alternative breakpoints involving exons 4, 5, or 7 have been reported but are comparatively rare.

Nevertheless, the formation of stable chimaeras is not always sufficient to yield a functional protein. For instance, *ESR1-NOP2* is translated into a stable hybrid product; however, the protein is transcriptionally inactive as it does not induce endocrine resistance or metastasis-associated processes [18]. Similarly, *ESR1-GYG1*, *ESR1-TCF12*, *ESR1-ARID1B* and *ESR1-PCMT1* seem non-functional as they fail to promote endocrine therapy-resistant growth in vitro, confirming that not all fused proteins lead to oncogenic effects [19]. This is likely because linking together gene fragments does not guarantee correct folding of the native protein domains or conservation of protein functionalities. Additional factors, such as domain structure and the biological role of the partner gene may be further implicated in creating fusions that act as oncogenic drivers, which are discussed further below.

Notably, out-of-frame fusions are not necessarily absent at the transcript level. RNA sequencing has frequently detected these events as fusion loci can produce multiple transcript isoforms through alternative splicing, variable breakpoints, or differential exon usage. Consequently, out-of-frame transcripts may coexist with in-frame isoforms that are overlooked when analyses focus on a single dominant fusion junction. Although these transcripts can be expressed, out-of-frame fusions are generally less likely to act as oncogenic drivers, as they are predicted to encode truncated or misfolded proteins that may be unstable, rapidly degraded, or functionally inert. Interestingly, some out-of-frame fusions can still contribute to oncogenesis, although indirectly. For example, *ESR1-TCF12* and *ESR1-ARID1B* do not activate ER signalling but appear resistant to ER degraders [19, 26], suggesting alternative mechanisms of action. This may be possible through disruption of tumour suppressor function or dominant-negative mechanisms, whereby the intact DNA-binding domain (DBD) of the fusion protein competes with the wild-type ER for DNA binding [2, 20]. Therefore, while generally considered non-functional, such fusions might still perturb critical pathways in subtle or context-dependent ways that need further exploration.In summary, the functional variability of *ESR1* fusions could be partly explained by the structural compatibility of the involved genes. This raises the possibility that the location of the fusion breakpoint is not random but rather driven by selective pressure to preserve the reading frame and generate functional, oncogenic proteins. In-frame fusions are more likely to act as oncogenic drivers, especially when they retain essential ER domains that contribute to enhanced signalling activity. Conversely, out-of-frame fusions are more likely to be passengers but may still disrupt tumour suppressor activity or alter cellular signalling pathways. Therefore, distinguishing between in-frame and out-of-frame rearrangements is crucial for identifying which fusions are more likely to be oncogenic and the mechanisms behind their biological activity.

## The role of fusion partners in *ESR1* fusion functionality

The *ESR1* gene has been found to fuse with nearly 30 distinct genes, creating a diverse set of chimeric proteins with a wide range of structural and functional consequences. While the *ESR1* portion is the primary driver of tumourigenic potential, the functional domains and biological roles of the partner gene may also influence the extent of these effects, by either enhancing or limiting ER-driven transcriptional activity. This is particularly evident from experiments showing that removal of the *ESR1* LBD/AF2 without replacement by a partner gene, abolishes transcriptional hyperactivity, indicating that the fusion partner contributes to the overall functionality [20, 26]. This raises the question: could the identity and properties of the partner gene be the key determinant that distinguishes weak from highly potent oncogenic *ESR1* fusions?

**Fig. 3:**
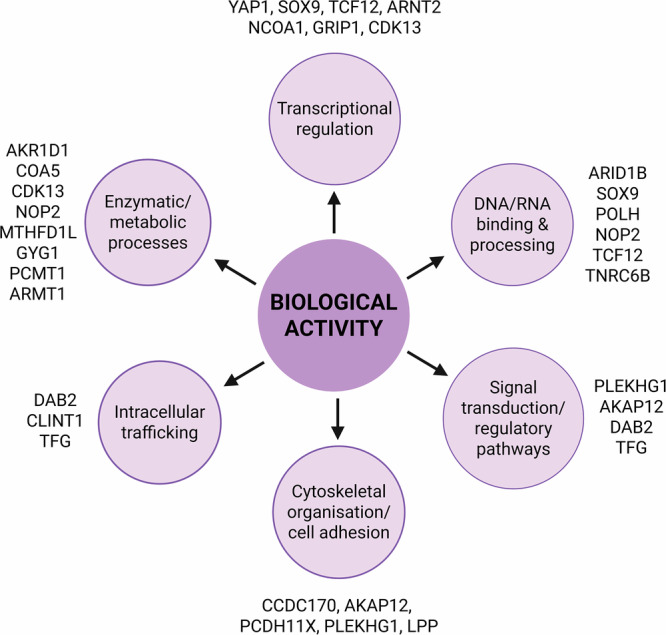
Biological functions of *ESR1* fusion partners. Functional annotation of *ESR1* fusions partners revealed six main functional categories: 1) transcriptional regulation, 2) DNA/RNA binding and processing, 3) signal transduction, 4) cytoskeletal organization and cell adhesion, 5) intracellular trafficking, and 6) enzymatic and metabolic activities. These diverse partners contribute distinct functional domains to the chimeric protein, potentially influencing localisation, transcriptional activity and various signalling cascades. Illustration created on BioRender.com.

Analysis of *ESR1* fusion partners (Fig. 3) reveals that many are involved in transcriptional regulation, DNA/RNA binding, and hormone signalling — all critical pathways commonly dysregulated in breast cancer. Frequently observed partners include *YAP1*, *SOX9*, *CDK13*, *POLH*, and *TCF12*, many of which are transcriptional regulators or components of signalling pathways such as the ERK1/2 cascade. Furthermore, several partners are enzymes with transferase activity (e.g., *ARMT1, MTHFD1L, NOP2*), or proteins involved in intracellular trafficking and scaffold functions (e.g., *AKAP12, GRIP1, DAB2*). Others, like *CCDC170* and *PCDH11X*, contribute to cytoskeletal organisation and cell adhesion, potentially affecting how cells respond to mechanical or extracellular cues.

Therefore, fusion partners also have the potential to influence the function of the resulting chimeric protein, as disrupting their native biological role could negatively impact their associated pathways. In some cases, oncogenic effects may arise not only from the activity of a chimeric fusion protein but also from inappropriate expression of the partner gene when placed under the control of *ESR1* regulatory elements. For example, when *CCDC170* fuses to *ESR1*, the truncated CCDC170 protein is expressed under the control of the *ESR1* promoter, leading to activation of downstream signalling pathways, such as AKT and ERK [17, 30, 50]. Aberrant CCDC170 expression promotes gain-of-function behaviours such as enhanced cell motility, anchorage-independent growth, and upregulation of scaffold proteins like Gab1, which is associated with invadopodia formation [17]. Collectively, these changes contribute to more aggressive tumour growth and resistance to anti-endocrine therapies, including oestrogen deprivation, tamoxifen, and fulvestrant [17, 20, 30, 50]. This mechanism contrasts with *ESR1* fusions that retain the ER DNA-binding domain and generate chimeric proteins capable of driving ligand-independent ER signalling. Distinguishing between fusions that act primarily through deregulated partner gene expression and those that function through constitutively active ER fusion proteins is therefore critical for interpreting their oncogenic mechanisms, therapeutic vulnerabilities, and relevance to endocrine resistance.

Overall, fusion partners may be doing more than passively stabilising the ER. The frequent involvement of transcription factors, signalling adaptors, and cytoskeletal regulators suggests a degree of selective pressure favouring fusions that reprogramme gene expression and survival pathways in a way that promotes more aggressive, therapy-resistant tumour phenotypes. This aligns with broader observations in cancer biology: while point mutations often affect DNA repair and cell cycle genes, gene fusions tend to involve regulators of transcription and signalling pathways involved in proliferation and homoeostasis [42, 46, 51, 52]. Consequently, future research should explore beyond the ERα component and instead draw more attention to how each fusion partner shapes the activity, localisation, and downstream effects of the chimeric protein. This may help predict oncogenic potential and identify candidates for targeted therapy, particularly for recurrent fusion partners.

## Domain architecture shapes *ESR1* fusion functionality

In addition to the native biological roles of the fusion partners, it is important to consider the domain composition of the genes involved. Most *ESR1* fusions retain several ER domains, while gaining new ones depending on the gene fused to it. For instance, many *ESR1* fusions retain or acquire nuclear localisation signals, which ensure that the chimeric protein is transported into the nucleus. This is essential for maintaining ER function, as its activity is predominantly nuclear. Moreover, the N-terminal DBD of ER is maintained in most *ESR1* fusions [15, 29], allowing the chimeric protein to potentially bind DNA at oestrogen response elements, and thus regulate gene expression. Similarly, many fused partners contribute their own DNA or RNA binding domains and transcriptional activation domains, which could further facilitate DNA binding and transcription; however, this requires experimental validation. The Activation Function-1 domain of ER is also generally conserved; however, the loss of the Activation Function-2 domain may alter the ability to recruit coactivators or corepressors, as these two domains are interconnected.

Nevertheless, the recruitment of transcriptional components may still be facilitated through the protein-interaction domains acquired from the fusion partners. Some examples observed in *ESR1* fusions include bHLH domains, PDZ domains and LxxLL motifs, which generally mediate interactions with other TFs. Additionally, some partner genes contain domains linked to their specific functions, such as CDK, ENTH, DH and PH domains, broadening the range of potential interactions and feeding into additional signalling pathways. This could explain the differences in tumourigenic potential, with some *ESR1* fusions promoting more aggressive behaviour and greater resistance to endocrine therapies than others.

The diversity in domain arrangements makes it challenging to establish which elements are responsible for their oncogenic properties. However, the recurrent retention or loss of specific protein domains suggests that certain genomic features may be preferentially selected during chromosomal rearrangements [42]. Conserved domains may be maintaining essential functionalities, such as DNA binding, transcriptional activation, nuclear localisation, or protein–protein interactions [1]. Conversely, consistent removal of domains, such as the LBD of *ESR1*, implies that their absence may be necessary to enable constitutive activity and bypass regulatory inhibition.

Therefore, understanding these domain configurations could help determine how the fusion operates and uncover potential therapeutic targets. Detailed structural and functional annotation of *ESR1* fusions will be essential to identify any partner-specific mechanisms of action that distinguish driver from passenger events.

## The role of intrinsically disordered regions in *ESR1* fusion functionality

The *ESR1* fusion breakpoints frequently localise around exon 6, sometimes at identical sites as observed with *ESR1-DAB2*, *ESR1-YAP1*, and *ESR1-GYG1*. This non-random distribution may be influenced by local sequence features or structural characteristics that make this region prone to recombination. Many fusions involve genes that are large and structurally complex, containing a few well-defined functional domains separated by long, uncharacterised regions [53]. These often correspond to intrinsically disordered regions (IDRs) — low-complexity amino acid sequences that lack a stable tertiary structure, allowing them to serve as flexible domains for diverse protein–protein interactions. Notably, proteins involved in translocations are frequently enriched in IDRs (43.3% vs 20.7% in the entire human proteome) [53], suggesting that intrinsic disorder may confer a functional advantage important for oncogenesis [2]. Consistent with this, breakpoints tend to cluster within disordered regions, avoiding disruption of functional protein domains [2, 53].

**Fig. 4:**
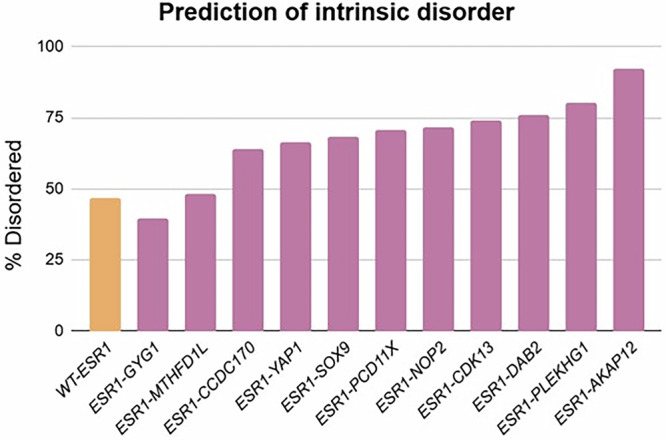
Prediction of intrinsic disorder in *ESR1* fusion proteins. The percentage of predicted intrinsically disordered regions (IDRs) was calculated for various *ESR1* fusion proteins and compared to wild-type (WT) *ESR1*. Predictions were performed using the VSL2 model in PONDR, which estimates the level of disorder based on amino acid sequence characteristics. This analysis highlights how fusion events may alter the disorder landscape of *ESR1*, potentially affecting the flexibility, interaction networks, and constitutive activity of the resulting chimeric proteins.

Computational predictions using PONDR (Fig. 4) show that all *ESR1* fusions apart from *ESR1-GYG1* are significantly more enriched in IDRs compared to wild-type *ESR1*. The extent of disorder varies depending on the fusion partner, suggesting that the partner gene largely determines the level of disorder introduced, given that the ER fragment is typically truncated at a consistent site. Interestingly, *ESR1* often fuses with genes that are inherently highly disordered, such as *AKAP12, PLEKHG1* and *DAB2*. PONDR predictions further support this, showing that *ESR1* rearrangements often eliminate most structured, globular domains and instead incorporate flexible, disordered segments from the partner gene. Furthermore, the fusion breakpoint usually occurs within such disordered segments, indicating potential structural weak points prone to chromosomal breakage.

The enrichment of IDRs in *ESR1* fusions may provide fusion proteins with a more flexible, dynamic structure that facilitates diverse protein–protein interactions and long-range communication [2]. This is particularly relevant for transcriptional regulators, where disordered regions may enhance interactions with DNA, co-factors, and chromatin regulators [53]. For example, the N-terminal transactivation domain of *ESR1*, which is critical for co-activator recruitment, is intrinsically disordered [54], reinforcing the functional importance of these regions.

IDRs may also facilitate phase separation – an emerging mechanism in cancer biology where proteins and nucleic acids condense into membraneless compartments. Oncogenic fusions such as *FET* and *NUP98* fusions exploit this property to form condensates that promote aberrant transcription programmes [55, 56]. This raises the possibility for *ESR1* fusions to act in a similar manner, producing condensates that amplify ER signalling. Additionally, intrinsic disorder may enable fusion products to evade degradation pathways that eliminate misfolded proteins by mimicking naturally flexible proteins [53].

Collectively, recurrent breakpoints, particularly around exon 6, point to the existence of genomic features that may predispose *ESR1* to rearrangement. However, further structural characterisation is needed in order to uncover any existing molecular signatures underlying this susceptibility. The consistent enrichment of IDRs in *ESR1* fusions suggests that while the *ESR1* portion drives many of the cancer-associated processes and therapy resistance, the fusion partner plays an equally important role by adding structural flexibility and potential new interaction sites, although their functional contribution remains to be explored. Investigating this could uncover novel therapeutic opportunities for fusion-driven cancers, such as targeting phase-separated condensates or disordered regions directly – an area of increasing interest in drug development.

## Therapeutic potential and future directions

Distinguishing driver fusions from neutral events remains a major challenge. To address this, large-scale sequencing coupled with functional studies is essential, as not all rearrangements produce stable or biologically active proteins. Improvements in detection methodologies are also needed to increase sensitivity, uncover new fusion variants, and better define their frequency and oncogenic relevance. Ultimately, fusion status may serve as a diagnostic or prognostic biomarker that helps refine tumour subclassification, predict therapy resistance, and guide treatment selection.

From a therapeutic perspective, *ESR1* fusions represent a challenging but promising target. Their structural variability and lack of LBD limit the effectiveness of conventional endocrine therapies, highlighting the need for drugs that target other conserved domains. For example, small molecules that target the DNA-binding domain (DBD) could block fusion proteins from binding to DNA, while approaches aimed at the N-terminal Activation Function-1 domain might disrupt co-factor recruitment and transcriptional activation. Alternative strategies could focus on indirectly suppressing fusion activity through downstream signalling pathways, such as CDK4/6 or PI3K/mTOR. Notably, previous work has demonstrated that CDK4/6 inhibition effectively suppressed cell proliferation driven by *ESR1-YAP1* and *ESR1-PCDH11X* fusions.

## Conclusion

*ESR1* fusions represent a rare but significant class of genomic alterations in breast cancer. Increasing evidence of their contribution to tumour behaviour and treatment response supports their role as potent oncogenic drivers that promote a more aggressive, metastatic phenotype. Their recurrent nature, consistent structural features, and enrichment in advanced, metastatic tumours suggest cancer specificity and functional importance. Many *ESR1* fusions produce chimeric proteins that retain DNA-binding capacity while losing ligand dependence, driving constitutive signalling associated with various pro-tumourigenic processes and therapy resistance. This recurrent rearrangement pattern may represent an adaptive evolutionary mechanism that allows tumours to bypass therapeutic pressure.

Nonetheless, their rare occurrence and variability in biological effects highlight that some *ESR1* fusions may be neutral, passenger events with limited pathological impact. Not all confer the same oncogenic potential or therapeutic vulnerability, suggesting that some may be random by-products of genomic instability. This functional diversity may be attributed to the role and composition of the partner gene involved, highlighting the need to explore partner contributions and potential off-target or dominant-negative effects. Furthermore, while certain fusions have been identified in multiple patients, others appear only once, raising questions about their biological relevance. Although they may represent random genetic variation, broader screening in larger cohorts, including non-malignant tissues, is needed to define their prevalence and clarify their role in the wider mutational landscape.

In conclusion, whether acting as oncogenic drivers or passenger events, *ESR1* fusions remain highly relevant in breast cancer, offering critical insight into the diverse mechanisms of therapy resistance, disease progression, and tumour evolution. Ongoing efforts to define their prevalence, biological functions, and therapeutic vulnerabilities are crucial to translate these findings into improved outcomes for patients with advanced, therapy-resistant breast cancer disease.
